# CT for estimating adequacy of lymph node dissection in patients with squamous cell carcinoma of the head and neck

**DOI:** 10.1186/s40644-021-00430-6

**Published:** 2021-11-21

**Authors:** Christiaan A. Rees, Joshua H. Litchman, Xiaotian Wu, Mariah M. Servos, Darcy A. Kerr, Ryan J. Halter, David A. Pastel, Joseph A. Paydarfar

**Affiliations:** 1 Geisel School of Medicine at Dartmouth, Hanover, NH USA; 2 Department of Radiology, Dartmouth-Hitchcock Medical Center, Lebanon, NH USA; 3 Thayer School of Engineering, Dartmouth College, Hanover, NH USA; 4 Department of Pathology and Laboratory Medicine, Dartmouth-Hitchcock Medical Center, Lebanon, NH USA; 5 Section of Otolaryngology, Dartmouth-Hitchcock Medical Center, Lebanon, NH 03756 USA

**Keywords:** 3D reconstruction, Computational modeling, Head and neck, Lymph node ratio, Lymph node yield, Neck dissection

## Abstract

**Background:**

Indices obtained from lymph node dissection specimens, specifically lymph node yield (LNY) and lymph node ratio (LNR), have prognostic significance in the setting of head and neck squamous cell carcinoma (HNSCCa). However, there are currently no validated tools to estimate adequacy of planned lymph node dissection using preoperative data. The present study sought to evaluate CT-derived estimates of lymphatic tissue volumes as a preoperative tool to guide cervical node dissection.

**Methods:**

Fifteen cervical lymph node dissections were performed in 14 subjects with HNSCCa. Preoperative CT-derived estimates of lymphatic tissue volumes were compared with gross pathology tissue volume estimates and pathologically-determined LNY.

**Results:**

Resected tissue volume (calculated using the triaxial ellipsoid method) correlates with CT-derived preoperative lymphatic volume estimates (*r* = 0.74, *p* = 0.003) while LNY does not(r = − 0.12, *p* = 0.67). When excluding pathologically enlarged lymph nodes (“refined” data), a negative correlation was observed between refined CT-derived volume estimates and refined LNY (*r* = − 0.65, *p* = 0.009).

**Conclusion:**

In the setting of cervical lymph node dissection, CT-derived lymphatic volume estimates correlate with resected tissue volume, but refined CT-derived volume estimates correlate negatively with refined LNY.

**Trial registration:**

Retrospectively registered.

**Level of evidence:**

4

## Background

Head and neck malignancies account for approximately 550,000 incident cases and 380,000 deaths worldwide annually [1]. The majority (approximately 90%) of head and neck malignancies are squamous cell carcinomas (HNSCCa), and it has been demonstrated that the presence of cervical nodal metastases is associated with reduced overall survival [2–4]. Furthermore, lymph node yield (LNY, the number of lymph nodes in a neck dissection specimen) and lymph node ratio (LNR, the number of cancerous nodes divided by the LNY) have been shown to have prognostic significance in the setting of HNSCCa [5–9]. Multiple large retrospective studies have shown a clear survival advantage in the setting of a higher LNY, including in elective neck dissection for the N0 neck [5–7, 10, 11]. However, because LNY and LNR are highly variable across individuals [8], it may be difficult for surgeons to determine whether an adequate lymph node dissection has been performed intraoperatively and for pathologists to anticipate the number of lymph nodes that a specimen is likely to contain. Since LNY varies due to patient-, surgeon-, and pathologist-associated factors, ‘*adequate sample*’ is challenging to define. However, recent studies suggest that LNYs of at least 16 to 26 nodes are associated with improved survival and/or reduced locoregional recurrence [12, 13].

Currently, there are no validated tools to estimate adequacy of planned lymph node dissection based on preoperative data, which has potentially important implications with respect to LNY and consequently prognosis. In this study, we test the hypothesis that CT-derived estimates of lymphatic tissue volume might serve as a preoperative tool to guide lymph node dissection. Specifically, we evaluate whether lymphatic volumes acquired on preoperative CT imaging correlate with the volume of lymphatic tissue removed intraoperatively as well as with LNY.

## Methods

### Selection of study subjects

This study was approved by the Committee for the Protection of Human Subjects at Dartmouth College (Study: 31330). The study population was composed of a consecutive cohort of patients with invasive squamous cell carcinoma (SCC) of the tonsil, tongue base, or supraglottic larynx who underwent neck dissections involving nodal levels II-IV in 2017–2019 by a single head and neck surgeon (JAP) as part of surgical management of their oropharyngeal or laryngeal primary tumor. Our criteria for performing level V neck dissection is very limited, therefore patients with level V neck dissection were not included Patients with and without node-positive disease were both included. Included patients had to have had a preoperative contrast enhanced CT scan. Patients with a history of prior surgery, radiation therapy, and/or chemotherapy were excluded.

### CT-derived estimates of lymphatic tissue volume

**Fig. 1:**
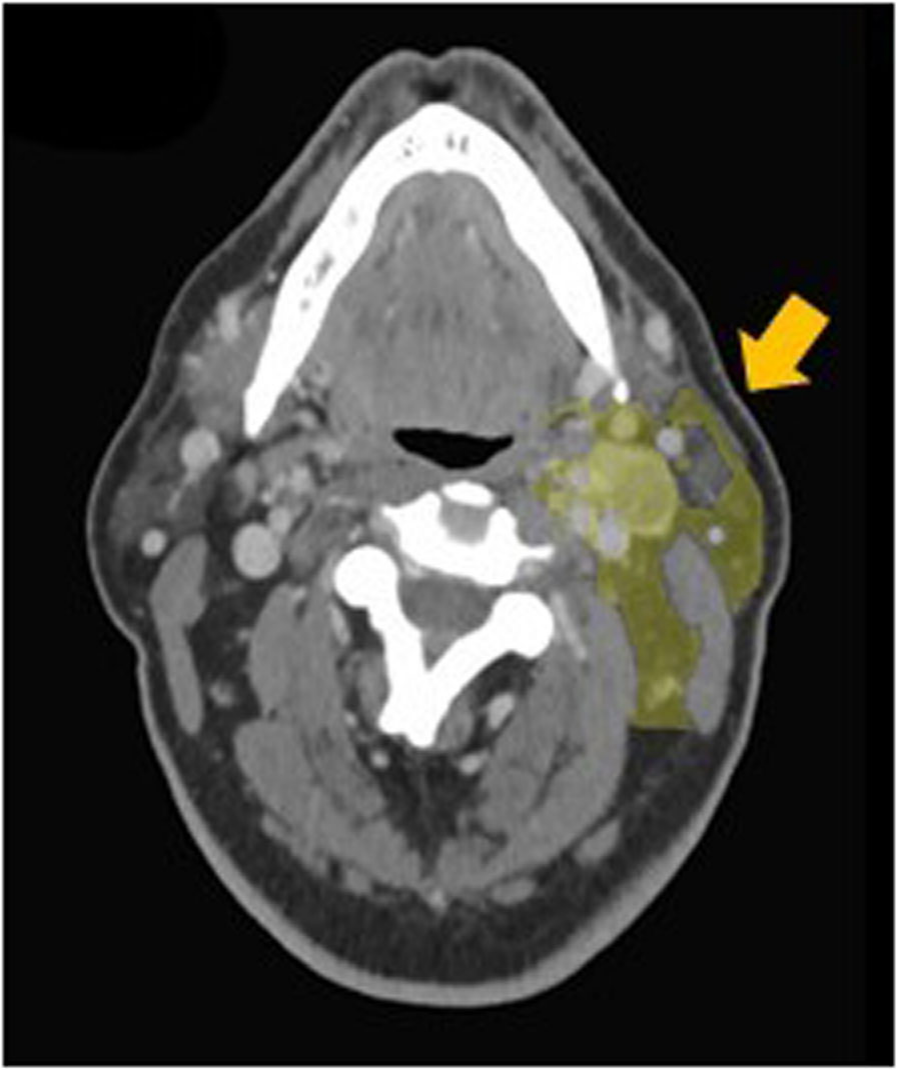
Segmented lymphatic tissue volumes on contrast-enhanced CT scans. Yellow highlighted regions correspond to the volume defined as level II-IV lymphatic tissue by Mimics following the processing steps described in Fig. 2

**Fig. 2:**
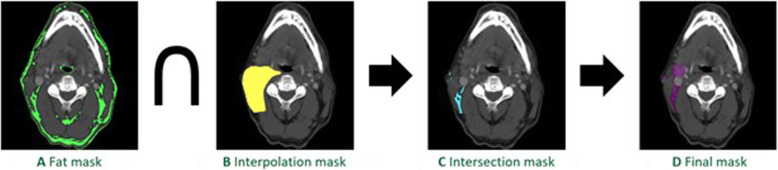
Segmentation protocol. **A** *Fat mask* based on tissue attenuation (green highlight). **B** *Interpolation mask* based on boundaries of nodal levels II–IV (yellow highlight). **C** *Intersection mask* generated from overlap of fat and interpolation masks (blue highlight). **D** Intersection mask edited to include lymphatic tissue and exclude vessels and lymphatic tissue outside the operative field to produce the *final mask* (purple highlight)

Neck lymphatic tissue volumes were estimated from preoperative contrast-enhanced CT scans using the imaging segmentation software Mimics (Materialise NV, Leuven, Belgium, Fig. 1). First, a volumetric model of the fat (fat mask) was generated based on Hounsfield units (HU range: − 205 to − 51, Fig. 2a). A second mask composed of nodal levels II–IV was generated by manually defining axial contours at 2.5 mm slice increments (or thinner, as needed) and then using software interpolation to fill in the volume (interpolation mask, Fig. 2b). Nodal boundaries were defined using the imaging-based nodal classification proposed by Som and colleagues with minor revisions in consultation with the performing surgeon (JAP) to ensure accuracy for each individual patient [14]. The two masks were intersected to create a volumetric model of the fat within nodal levels II–IV (intersection mask, Fig. 2c). This third mask was edited manually to ensure that it included any visualized nodal tissue and excluded vessels and lymphatic tissue outside the operative field (final mask*,* Fig. 2d). A duplicate mask was created from this final mask but with pathologically-enlarged lymph nodes (greater than or equal to 11 mm in minimal axial diameter) removed to perform additional analysis with CT-derived preoperative volumes that were not skewed by these enlarged lymph nodes (refined mask, not shown). The final and refined masks were reviewed for accuracy by a neuroradiologist (DAP) with 15 years of experience and by a head and neck surgeon (JAP) with over 18 years of experience. Though segmentation was mostly performed on the axial slices, the other two image planes (coronal and sagittal) were used for minor refinements.

### Processing and analysis of lymphatic tissue specimens

**Fig. 3:**
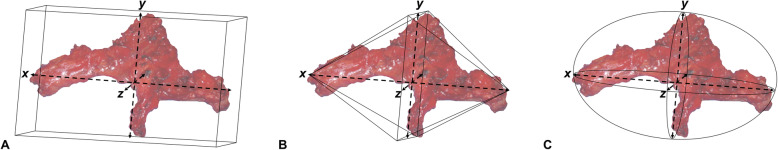
Example resected lymphatic tissue specimen. Resected pathological volume is estimated via three methods: a rectangular prism (*x y z*), a rectangular bipyramid ($\frac{2}{3}\ x\ y\ z$), and a triaxial ellipsoid ($\frac{\pi }{6}\ x\ y\ z$)

Surgical specimens included complete levels II-IV neck dissections with anatomic levels specified by the surgeon via attached sutures and those in which dissected levels II, III, and IV were submitted separately. The lymphatic tissue was processed per routine protocol in the pathology laboratory. In brief, the overall size of each gross specimen was measured in three dimensions (*x, y,* and *z*), and based on this, tissue volumes were estimated (the resected volume). Given that the lymphatic tissue specimens were irregularly shaped (Fig. 3), three different three-dimensional shapes were used to estimate lymphatic tissue volume: a rectangular prism (*x y z*), a rectangular bipyramid ($\frac{2}{3}\ x\ y\ z$), and a triaxial ellipsoid ($\frac{\pi }{6}\ x\ y\ z$).

For complete levels II-IV neck dissection specimens, each lymph node level was manually separated as a block of node-bearing fibroadipose tissue based on the location of the sutures. Each nodal level was then palpated for the presence of candidate lymph nodes, and all candidate lymph nodes were dissected from adjacent fibroadipose tissue. For grossly negative lymph node candidates, all candidate nodal tissue was submitted for histologic evaluation. For grossly positive lymph nodes, two to three representative sections from the periphery of each involved lymph node were submitted. The submitted tissue was fixed in 10% buffered formalin for 24–48 h at room temperature before automated tissue processing and embedding to create formalin-fixed, paraffin-embedded tissue blocks. Five-micron tissue sections were stained with hematoxylin and eosin. The slides were reviewed by one of three subspecialized head and neck pathologists (average years of experience: 25, range 11 to 40) for diagnosis and cancer synoptic reporting, including assessment of the number of lymph nodes removed and the presence or absence of tumor in each lymph node.

### Data processing and statistical analysis

The total estimated lymphatic tissue volumes from preoperative CT scans were exported directly from Mimics and referred to as the raw CT volume. Refined CT volumes were defined as the raw CT volume minus the volume contributed by pathologically enlarged nodes as defined above. LNY and estimates of resected tissue volume were obtained from review of the pathology reports. Spearman’s correlation coefficient was used to evaluate the relationship between variables, with both *r* (the strength of the association) and associated *p*-values reported. A *p*-value of 0.05 was used as the threshold for statistical significance. Depending on the characteristics of the data, figures are either presented in log-log or log-linear scale, although all statistical analyses were performed prior to log transformation.

## Results

### Baseline characteristics of study participants

Fourteen subjects meeting inclusion criteria were enrolled, including three females and 11 males (Table 1). One patient underwent bilateral neck dissections (Table 1, Patient 14). The average age of study subjects was 63 years (range: 49 to 85 years), and the average body mass index (BMI) was 28.1 kg/m^2^ (range: 21.5 to 36.1 kg/m^2^). Primary tumor sites consisted of base of tongue (*n* = 9), tonsil (*n* = 4), and supraglottic larynx (*n* = 1). Left-sided dissection was performed in nine subjects, right-sided dissection in four, and bilateral dissection in one. Following pathological staging, 11 subjects had evidence of nodal involvement (N1 or N2b), and 12 had human papillomavirus-positive (HPV+) primary tumors. All of the patients’ preoperative CT used in this study had parameters listed in Table 2.

**Table 1:** Characteristics and lymph node information of study participants

Patient	Age	Sex	BMI	Primary Site	Pathologic Stage^a^	HPV (+/−)	Dissection Laterality	Lymph Node Yield (LNY)	Refined Lymph Node Yield	Pathologically Positive Lymph Nodes
1	57	M	29	Tonsil	T1N2bM0	+	Left	30	27	4
2	85	F	22	Base of tongue	T1N0M0	–	Left	26	26	0
3	60	M	26	Base of tongue	T2N1M0	+	Right	20	18	1
4	49	M	31	Base of tongue	T2N1M0	+	Right	41	39	1
5	54	M	28	Base of tongue	T1N1M0	+	Left	65	64	1
6	62	M	27	Base of tongue	T1N1M0	+	Left	33	32	1
7	72	M	24	Base of tongue	T2N2bM0	+	Right	44	42	2
8	66	M	27	Tonsil	T1N1M0	+	Left	23	21	1
9	70	M	36	Base of tongue	T2N0M0	+	Right	15	15	0
10	65	F	27	Base of tongue	T2N1M0	+	Right	44	42	1
11	77	M	23	Tonsil	T1N1M0	+	Left	23	23	1
12	53	M	29	Base of tongue	T1N2bM0	+	Left	33	31	2
13	49	M	33	Tonsil	T2N1M0	+	Left	26	26	0
14	66	F	31	Supraglottic larynx	T3N0M0	N/A	Bilateral	43 L/35R	43 L/35R	0/0

^a^All staging based on AJCC 7th Edition

**Table 2:** Parameters for preoperative CT scans

Patient	Manufacturer	Model	Slice Thickness (mm)	kVp	mAs	convolution kernel/algorithm
1	TOSHIBA	Aquilion	2	120	119	FC05
2	GE MEDICAL SYSTEMS	LightSpeed VCT	2.5	120	120	STANDARD
3	GE MEDICAL SYSTEMS	LightSpeed16	1.25	120	477	STANDARD
4	GE MEDICAL SYSTEMS	LightSpeed16	1.25	120	477	STANDARD
5	TOSHIBA	Aquilion	3	120	150	FC04
6	SIEMENS	SOMATOM Definition AS	1.5	100	283	I31f\3
7	SIEMENS	SOMATOM Definition AS	3	100	185	B31s
8	TOSHIBA	Aquilion	3	120	250	FC09
9	TOSHIBA	Aquilion ONE	3	120	50	FC08
10	TOSHIBA	Aquilion	3	120	25	FC08
11	GE MEDICAL SYSTEMS	LightSpeed VCT	0.625	120	120	STANDARD
12	GE MEDICAL SYSTEMS	LightSpeed VCT	0.625	120	281	STANDARD
13	TOSHIBA	Aquilion PRIME	1	120	231	FC08
14	TOSHIBA	Aquilion	2	120	71	FC08

### Estimates of lymphatic tissue volume using preoperative CT imaging

The average CT-derived estimate of lymphatic tissue volume (raw CT volume) was 38.6 cm^3^ (range: 11.5 to 78.6 cm^3^). After manual removal of pathologically-enlarged lymph nodes on imaging, the average preoperative lymphatic tissue volume (the refined CT volume) decreased to 30.0 cm^3^ (range: 11.5 to 48.6 cm^3^).

### Estimation of resected volume

The average resected volume across all specimens was 127.9 cm^3^ (range: 19.4 to 413.8 cm^3^) using a rectangular prism estimate, 85.3 cm^3^ (range: 12.9 to 275.9 cm^3^) using a rectangular bipyramid estimate, and 67.0 cm^3^ (range: 10.2 to 216.7 cm^3^) using a triaxial ellipsoid estimate. Resected volume using the rectangular prism, rectangular bipyramid, and triaxial ellipsoid estimate were strongly correlated with raw CT volume (*r* = 0.74, *p* = 0.003 for all three estimates). Resected volume estimates were, on average, greater than CT-derived volume estimates using all three methods, although the triaxial ellipsoid estimates were the most accurate. The relative volume estimates (resected volume estimate divided by CT-derived volume estimate) were: 3.1 (range: 0.5 to 10.0) using the rectangular prism estimate, 2.1 (range: 0.3 to 6.7) using the rectangular bipyramid estimate, and 1.6 (range: 0.2 to 5.3) using the triaxial ellipsoid estimate.

### Correlation of lymph node yield with CT volumes

**Fig. 4:**
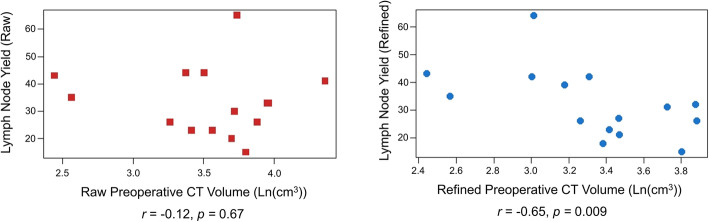
Log-linear plots depicting the relationship between resected CT volume (*x-*axis) and lymph node yield (*y*-axis) for both raw (left, red) and refined (right, blue) CT-derived estimates. For raw data: *r* = − 0.12, *p* = 0.67. For refined data: *r* = − 0.65, *p* = 0.009

The LNY was determined from pathological analysis. Average LNY was 33.4 (range: 15 to 65). LNY was compared with the CT-derived volume estimates. LNY was not significantly correlated with raw CT volume (*r* = − 0.12, *p* = 0.67). In contrast, refined LNY, which excluded the pathologically enlarged nodes noted on CT (average: 32.3, range: 15 to 64), was negatively correlated with refined CT volume (*r* = − 0.65, *p* = 0.009). The relationship between LNY and CT volume for raw and refined estimates is presented in Fig. 4.

## Discussion

This study involving 15 level II-IV neck dissections in 14 patients demonstrates that: 1) CT-derived estimates of raw CT volume was correlated with actual resected lymphatic tissue volume, as estimated by surgical pathology, although resected estimates are larger than CT-derived estimates; 2) Overall LNY was not correlated with raw CT volumes; 3) Refine LNY (where pathologic nodes are removed) is negatively correlated with CT-derived estimates of refined CT volume. To the best of our knowledge, this is the first attempted use of CT imaging to estimate resected lymphatic tissue volume and LNY and may represent a first step towards the development of a novel tool to guide lymphadenectomy.

The goal of this study was to test the hypothesis that CT-derived lymphatic tissue volume estimates correlate with pathologic volumes and could accurately predict LNY. Some authors have shown that the extent of neck dissection and/or number of neck levels excised is positively associated with LNY [15, 16] suggesting that resected tissue volume may be correlated with LNY. We used segmented CT-derived lymphatic tissue volume to estimate the resected volume during neck dissection and were able to demonstrate that these segmented volumes do correlate with gross pathologic volume estimates. We hypothesized that the use of preoperative CT imaging to estimate the amount of lymphatic tissue that should be removed to achieve a certain LNY could substantially impact decision making and quality in the surgical management of head and neck cancer. For instance, if based on preoperative CT it could be established that a sufficient LNY would be achieved without dissecting level 4 in the left neck, this would reduce injury potential to the thoracic duct. On the other hand, preoperative imaging could suggest that, to achieve a sufficient LNY, a more extensive neck dissection would be required. This might prompt further patient counselling or even an alternate non-surgical treatment strategy. Furthermore, if an expected yield based on preoperative imaging is not achieved at the time of surgery, this may prompt a quality review to ensure that proper surgical technique is being employed or that procedures for processing of pathologic specimens are being appropriately followed.

**Fig. 5:**
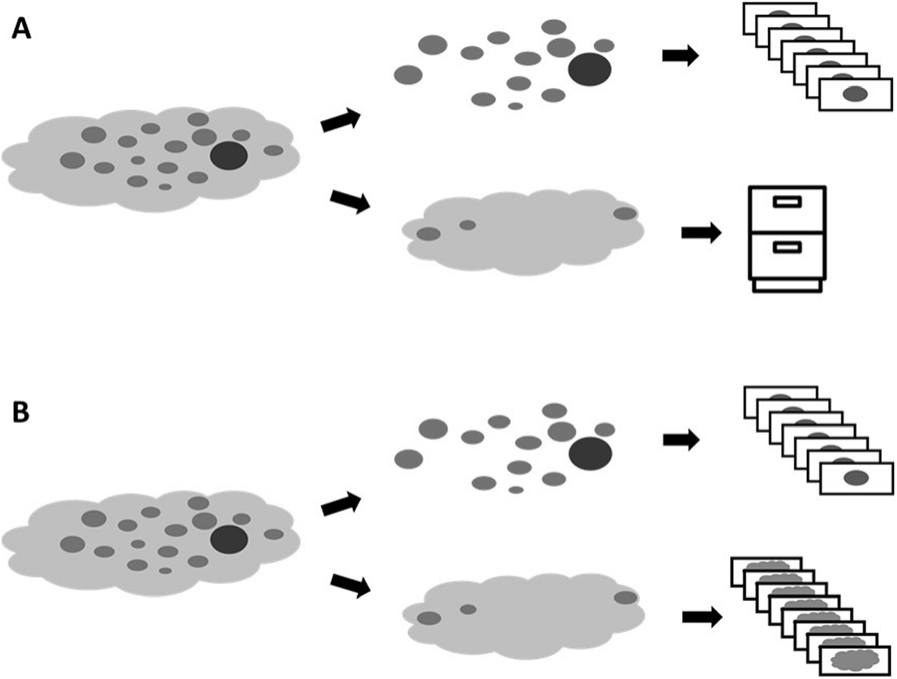
(A) Current neck dissection pathologic processing protocol. Candidate lymph nodes in specimen are separated out by palpation and examined histologically. Most of the remaining adipose tissue is stored for future processing as needed and therefore this volume of tissue is not accounted for. (B) Revised protocol based on the findings of Holcomb et al. [15] In this protocol all additional fatty tissue is processed thereby accounting for the entire specimen volume

Although a correlation between CT-derived neck dissection volumes and LNY was not established in this study, our findings do raise important questions regarding confounding factors which may have influenced these results. One possible explanation could be that the standard approach for processing of pathologic specimens targets only the manually identified candidate lymph nodes for histological evaluation rather than microscopically assessing the total volume of tissue removed. As illustrated in Fig. 5A, this technique results in a certain amount of adipose tissue that is not analyzed but would be included in any volumetric measurements and could contain small grossly unidentified lymph nodes. Interestingly, we did identify a negative correlation between LNY and refined CT volumes (i.e., CT volumes in which known pathologic nodal volume was subtracted). The observation that LNY is negatively correlated with refined CT volume is arguably counterintuitive, as a greater lymphatic tissue volume might reasonably be expected to contain a greater total number of lymph nodes. It is unclear whether this finding represents a biological phenomenon or is an artifact of sample processing. A recent report by Holcomb et al [15] supports this latter concept. In this study the authors reported on a pathology protocol modification where once all visible nodes were removed from the lymphadenectomy specimen, instead of storing the residual adipose tissue, this material was also placed in cassettes for processing and analysis (Fig. 5B). The authors found a 30% increase in LNY yield which was significant on multi-variate analysis. Other factors which could have affected the results in this study include the skill and experience of the prosector and patient factors such as age and BMI.

With respect to study limitations, we acknowledge that the small sample size may have reduced our statistical power to identify associations between variables of interest and limited our ability to utilize more advanced statistical approaches that could have allowed us to evaluate the influence of covariates such as age, gender, or BMI on our comparisons of interest. We also acknowledge that despite the strong correlation between resected volume and raw CT-derived volume estimates, resected volume estimates are highly variable depending upon the method used. We suspect that this is because of the irregular shape of the lymphatic tissue specimen excised, which is not accounted for by relatively crude resected volume estimates. Due to the retrospective nature of this study, more precise tissue volumetric measurements could not be performed.

A prospective study, currently underway aims to examine tissue volumes at the time of neck dissection which will be correlated with CT-derived volumes of the same nodal station. Furthermore, pathologic specimen processing as outlined by Holcomb et al [15] has been initiated in order to capture the entirety of the neck dissection specimen. Other areas of investigation would include developing more efficient methods of segmentation of the nodal basins of interest utilizing machine learning algorithms [17].

## Conclusions

CT-derived lymphatic tissue volumes estimate resected volume during neck dissection, but LNY did not correlate with these estimates. However, when excluding the volume of pathologically-enlarged lymph nodes visible on pre-operative CT imaging, a negative correlation was observed between these refined CT-derived lymphatic volumes and LNY, suggesting that the presence of pathologically enlarged lymph nodes within neck dissection specimens may decrease lymph node yields.

## Data Availability

The datasets used and/or analyzed during the current study are available from the corresponding author on reasonable request.

## Abbreviations

HNSCCa: Head and Neck Squamous Cell Carcinoma
LNY: Lymph Node Yield
LNR: Lymph Node Ratio
HPV: Human Papilloma Virus
